# Advanced Sampling Technique in Radiology Free-Text Data for Efficiently Building Text Mining Models by Deep Learning in Vertebral Fracture

**DOI:** 10.3390/diagnostics14020137

**Published:** 2024-01-08

**Authors:** Wei-Chieh Hung, Yih-Lon Lin, Chi-Wei Lin, Wei-Leng Chin, Chih-Hsing Wu

**Affiliations:** 1 Department of Family and Community Medicine, E-Da Hospital, I-Shou University, Kaohsiung 82445, Taiwan; ed107698@edah.org.tw (W.-C.H.); ed104283@edah.org.tw (C.-W.L.); ed106587@edah.org.tw (W.-L.C.); 2 School of Medicine, I-Shou University, Kaohsiung 84001, Taiwan; 3 Institute of Biotechnology and Chemical Engineering, I-Shou University, Kaohsiung 84001, Taiwan; 4 Department of Computer Science and Information Engineering, National Yunlin University of Science and Technology, Douliu 64002, Taiwan; yihlon@yuntech.edu.tw; 5 Institute of Gerontology, College of Medicine, National Cheng Kung University, Tainan 70101, Taiwan; 6 Department of Family Medicine, National Cheng Kung University Hospital, College of Medicine, National Cheng Kung University, Tainan 70101, Taiwan

**Keywords:** sampling method, free-text data, vector sum, vertebral fracture, radiology report

## Abstract

This study aims to establish advanced sampling methods in free-text data for efficiently building semantic text mining models using deep learning, such as identifying vertebral compression fracture (VCF) in radiology reports. We enrolled a total of 27,401 radiology free-text reports of X-ray examinations of the spine. The predictive effects were compared between text mining models built using supervised long short-term memory networks, independently derived by four sampling methods: vector sum minimization, vector sum maximization, stratified, and simple random sampling, using four fixed percentages. The drawn samples were applied to the training set, and the remaining samples were used to validate each group using different sampling methods and ratios. The predictive accuracy was measured using the area under the receiver operating characteristics (AUROC) to identify VCF. At the sampling ratios of 1/10, 1/20, 1/30, and 1/40, the highest AUROC was revealed in the sampling methods of vector sum minimization as confidence intervals of 0.981 (95%CIs: 0.980–0.983)/0.963 (95%CIs: 0.961–0.965)/0.907 (95%CIs: 0.904–0.911)/0.895 (95%CIs: 0.891–0.899), respectively. The lowest AUROC was demonstrated in the vector sum maximization. This study proposes an advanced sampling method, vector sum minimization, in free-text data that can be efficiently applied to build the text mining models by smartly drawing a small amount of critical representative samples.

## 1. Introduction

In medical practice, there are many types of data, including numeric, categorical, or textual, and the numeric and categorical data can be processed directly in statistical analysis and have various clinical applications in patient care [1]. Textual data such as radiology free-text reports were commonly recorded by physicians from free typing with possibly high variability in the data between different physicians. It was necessary to manually review each report to further utilize the data; in addition, this process is inefficient and complex, with potentially high error rates [2,3]. Natural language processing (NLP) is a branch of artificial intelligence (AI) that makes a computer understand, analyze, and interpret human language through machine learning and could be a solution for extracting critical information from free-text data. NLP is widely applied in many systems including search engines, voice assistance, translation software, and chatbots [4]. We can extract vital information from free-form text, known as unstructured data, by NLP, and this process is called data mining, also known as semantics in machine learning [5]. Building the semantic text mining model through the NLP technology seemed to be an alternative way for applying free-text data in medicine [6,7].

Vertebral compression fracture (VCF) is a common disease in the aging population, significantly impacting quality of life and associated with higher mortality [8,9]. The prevalence of VCF is around 10–20% in 50-year-old women and increases to 30–50% in 90-year-olds worldwide [10]. Not only in postmenopausal women, VCF is also a vital health issue for older men [11]. Prior osteoporotic VCF is correlated with five times greater risk of subsequent VCF within a year and other osteoporotic fractures including hip fractures [12,13]. The identification and management of VCF is crucial for reducing morbidity due to refracture, disability or chronic pain, and mortality [13]. Capture the Fracture (CTF)^®^, projected by the International Osteoporosis Foundation (IOF), is a program to implement case management models, known as fracture liaison services (FLS), with 13 standards to prevent secondary fractures [14]. The Standard 4 of CTF^®^ is to identify the patients who are reported by the radiologists to have VCF on medical imaging exams and deliver further evaluation and treatment for them [14]. Most of the reports for medical imaging exams by radiologists are in the free-text form, and identifying patients with VCF from the reports is needed to be executed by manual reviews from clinical staff. Obviously, these works on VCF require high manpower and are underdiagnosed [15]. Semantic text mining models could be built to efficiently identify the reports with the semantics of VCF from the radiology free-form text reports of X-rays by supervised machine learning [16]. The barriers in implementing FLS, including high manpower demand and a high underdiagnosed rate for identifying the patients with VCF [17,18], can be significantly improved by applying semantic text mining in healthcare institutions. In addition, a supervised machine learning algorithm has been designed to train the machine using well-labeled samples, which were tagged with the correct answers and called the training set [19]. It is important but time-consuming to identify which clinical staff gave the labels, with or without VCF, to some of the radiology text reports and then build the semantic text mining models by supervised machine learning. These newly designed models could be used for identifying VCF from other radiology reports without labels.

For building the models, manually labeling for the partial data to become the role as the training set was still the critical step before processing machine learning with two important difficulties [18,20]. First, the labeling performed by clinical physicians was also time-consuming owing to recoding [20,21]. Next, it was difficult to determine which part of the data should be chosen to be the training set from all the studied data, and this step may have significantly influenced the predictive effect of the following building models. If the training set data was biased, for example, the data did not cover the description of “biconcave fracture”, the semantic text mining models for VCF building in this situation could not properly identify the text reports with “biconcave fracture” as VCF was detected positive [1,22]. Sampling the small amounts of critical representative data to be the training set for building the models was an efficient way; however, there was no credible sampling method in free-text data, different from numerical data that can be sampled by random, stratified, systemic, or cluster methods [23]. With the reliable sampling process, efficiently building the semantic text mining models for extracting the information with a high accuracy rate from not only radiology reports but also other textual data such as the reports of esophagogastroduodenoscopic, electrocardiographic, and sonographic exams, could be achieved. The aim of this study is to establish the advanced sampling methods in free-text data for efficiently building the semantic text mining models by deep learning while identifying VCF from radiology free-text reports as exemplars.

## 2. Materials and Methods

### 2.1. Database

This study enrolled 30,102 free-text radiological reports for X-rays from experienced radiologists in the database of E-Da Hospital from 1 August 2018 to 31 January 2020. The hospital serves approximately 1,000,000 (outpatients)/40,000 (inpatients) people/year. It is a tertiary referral hospital in southern Taiwan [24]. The inclusion criteria are X-ray examination of the lateral view of the thoracolumbar or lumbosacral spine, and text reports must be written in English. After excluding text reports with Chinese–English mixed communication (*N* = 803), and duplicate data (*N* = 1898), a total of 27,401 X-ray reports were analyzed.

### 2.2. Labeling of Vertebral Compression Fracture

After reading the reports, all data were labeled by two experienced clinical physicians for whether a case is VCF positive or not. The labels only determined the semantics in textual reports without reviewing X-ray image data or the consideration of the correctness of the reports from the radiologists. The possibly relevant descriptions for determining VCF included compression fracture, anterior wedging, biconcave fracture, biconcave deformities, crush fracture, decreased vertebral height, insufficiency fracture, osteoporotic vertebral fracture, concave deformity, vertebral collapse, and similar statements. If the labels given by the two clinical physicians are different, the third experienced clinical physician is needed to give labels and discuss with the first two physicians again until the three physicians make the labels consistent.

### 2.3. Sampling Methods

We performed data sampling by four methods, including vector sum minimization, vector sum maximization, stratified sampling, and simple random sampling, by an arbitrary fixed percentage, including 1/10, 1/20, 1/30, and 1/40 of the total number of radiology text reports enrolled in this study, to prepare the training set for building semantic text mining models. The method was initially executed using the document to vector technology that proposed the neural network language model to convert each text report into a 400-dimensional vector, where the 400 value was used to represent one report [25]. Similar semantic documents had similar vector presentation (e.g., the report of X-ray involved the meaning of “VCF” is close to “vertebroplasty”). Each radiology text report included in this study was recognized as one sample. Based on the vector of each text report, all samples were clustered by hierarchical agglomerative clustering (HAC), an unsupervised learning algorithm to group subjects based on their similarity like vector presentation, and each group had its own cluster center [26]. The setting used in HAC that was (1) Euclidean distance, known as straight line distance between two points in Euclidean space [27], was at or less than 35 between the sample in the group and its cluster center, and (2) the window size that was the maximum distance between the focus word and its contextual neighbors was set to 6 (consider six words in front of and behind the word). The vector sum minimization sampling method was to minimize the vector sum of all the drawn samples relative to the cluster center in each group. Contrary to the first method, the second method maximized the vector sum of all the drawn samples relative to the cluster center in each group. The stratified sampling randomly sampled data in each group according to the required ratio. The previous three methods were delivered by drawing samples from each group, clustered by HAC, according to a fixed percentage. The fourth method used simple random sampling without clustering.

### 2.4. Machine Learning Methodologies and Statistical Analyses

The machine learning algorithms and statistical analyses were performed using Python (version 3.7.3; Python, Beaverton, OR, USA) for Windows. The validation and comparison of the effects between the models were carried out using MedCalc (version 19.2.0; MD aware LLC, San Francisco, CA, USA). The first three sampling methods clustered by HAC, the vector sum of all drawn samples relative to the cluster center in each group was calculated and expressed by Euclidean distance shown as mean ± standard deviation. The Kruskal–Wallis test was used to analyze the Euclidean distance of all clusters produced by different sampling methods.

**Figure 1:**
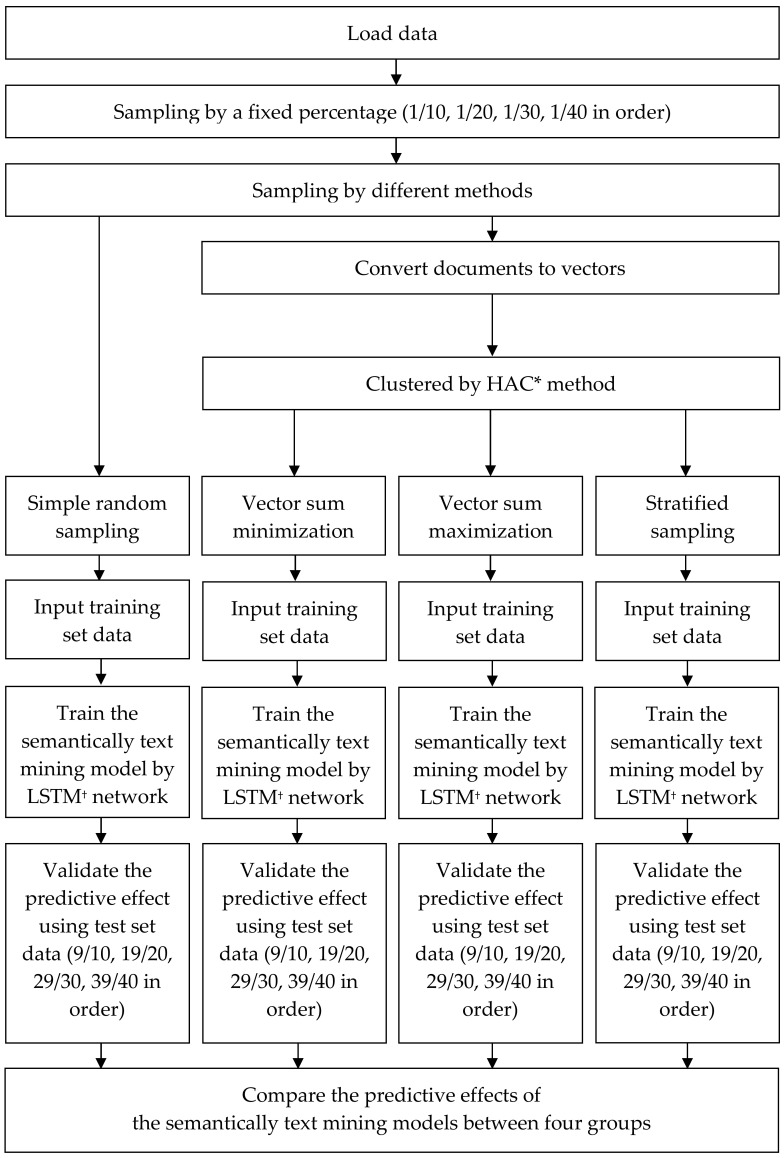
Flowchart of sampling, building, and validating the text mining models. * HAC: hierarchical agglomerative clustering. † LSTM: long short-term memory.

The flowchart of sampling data, and building and validating semantic text mining models is shown in Figure 1. The samples drawn by the aforementioned sampling methods were applied as the training set for building semantic text mining models using a long short-term memory (LSTM) network, a deep recurrent neural network architecture capable of processing sequential data with order dependence such as NLP [28]. The settings used in model building using LSTM were as following:

- Word lengths of the text report data: 200.
- Dimensions of the word vectors: 100.
- Window size: 6 (Supplementary Figure S1).
- Input neurons: 20,000 (200 words × 100 dimensions), and the input data of each neuron was the value of word vectors.

Additionally, the following settings were applied:

(1) Activation function: sigmoid.
(2) Optimizer: Adam with a learning rate of 0.001.
(3) Number of layers: 5.
(4) Loss function: binary_crossentropy

After the model was established, other remaining samples not in the training set were used to validate the predictive effects of the models, called the test set. When sampling 1/10 of the total number of radiology text reports as the training set, the remaining 9/10 data, which was not in the training set, was used in the test set. Following this rule, when the percentage 1/20, 1/30, 1/40 was applied as the sampling percentage to become the training set, 19/20, 29/30, 39/40 of the other remaining data were used as the test set, respectively. The effects of the semantic text mining models were presented by area under the receiver operating characteristics (AUROC) and the DeLong test was applied to compare the AUROC of two models [29]. The receiver operating characteristics curve is a graph formed using two parameters, true positive rate and false positive rate, to demonstrate the performance of a classification model at various thresholds settings. AUROC, the entire two-dimensional AUROC, represents the degree or measure of separability ranging from 0 to 1 [30]. The value of AUROC is 1 in the model whose predictions are 100% and 0 in the model whose predictions are 0% [31]. In this study, four sampling methods and four sampling percentages were implemented to establish 16 models. The predictive effects of the semantic text mining models were validated. Unless otherwise stated, the data were expressed as the percentage for categorical variables and the mean of the standard deviation for continuous variables. Analysis of variance was used to analyze continuous variables. A *p* value of ≤0.05 was defined as statistically significant.

## 3. Results

### 3.1. Baseline Characteristics of the Data

The mean age of the examinees of X-rays were 69.1 ± 12.5-year-olds with 30.8% (*N* = 8440) males. The X-ray examinations were 14,386 in thoracolumbar spine (52.5%) and 13,015 in lumbosacral spine (47.5%). The reports labeled as VCF data were 11,155 (40.7%). There are four sampling methods in this study and the first three methods were performed by HAC to divide into 46 groups according to the document vectors. The schematic of the clustering by HAC with embedding in two dimensions is shown in Supplementary Figure S2. The amount of data was 596 ± 561, and the amount of data with VCF was 243 ± 221 in each group (Table 1). The value of the vector sum presented by Euclidean distance in the sampling methods of vector sum minimization, vector sum maximization, or stratified sampling were 2.9 ± 2.3, 19.5 ± 26.1, or 12 ± 10.3 (*p* < 0.001) when the sampling percentage was 1/10 (Table 1). The statistical analysis revealed that the lowest vector sum occurred in the sampling method of vector sum minimization, the second lowest in stratified sampling, and the highest in vector sum maximization. The same results were also demonstrated in the other three sampling ratios: 1/20, 1/30, and 1/40 (Table 1).

### 3.2. Model Performance across Different Sampling Method

**Figure 2:**
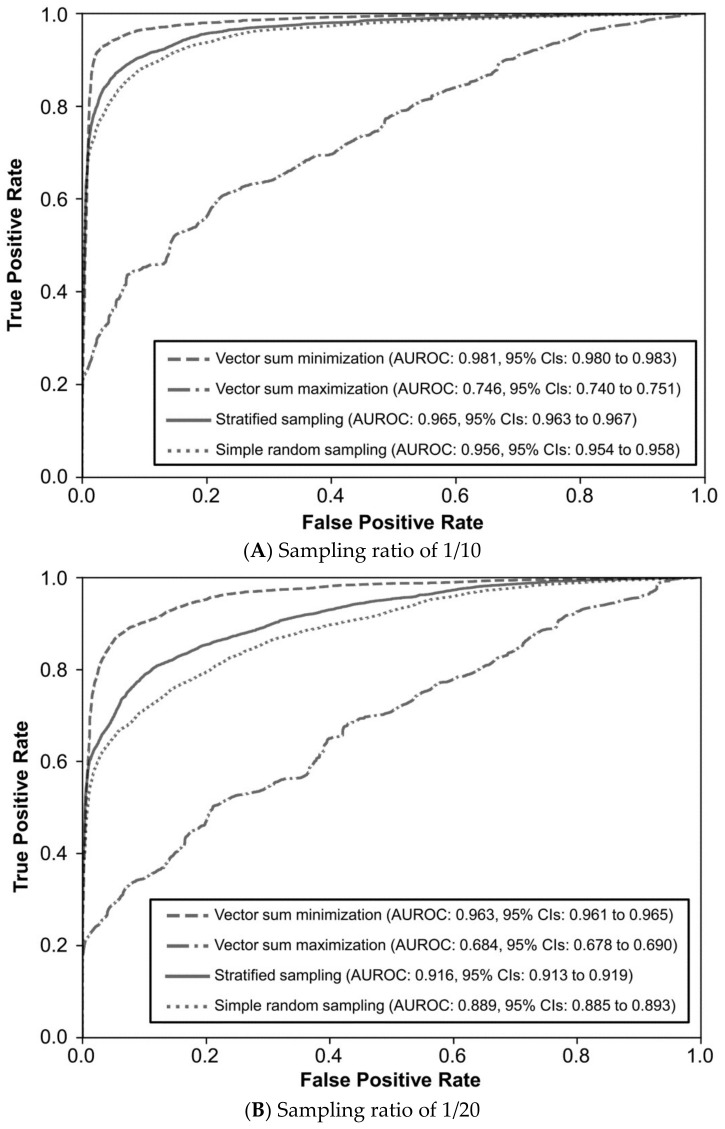

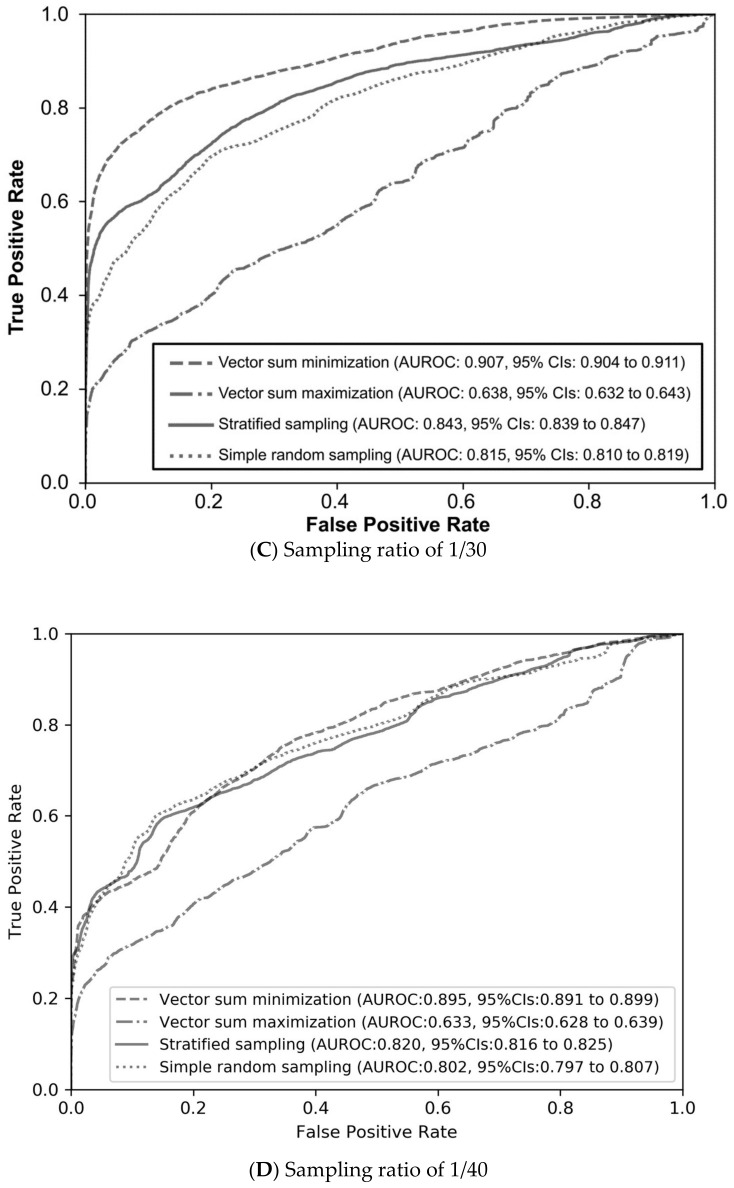
Predictive effect of semantic text mining models built by the samples drawn via different sampling methods were shown in AUROC (**A**) sampling ratio of 1/10, (**B**) sampling ratio of 1/20, (**C**) sampling ratio of 1/30, and (**D**) sampling ratio of 1/40. AUROC: area under the receiver operating characteristics. 95% CIs: 95% confidence intervals. All the *p* value is < 0.001 between any two sampling methods at four sampling ratios.

The predictive effects of each semantic text mining model built by the different training sets drawn in different sampling methods and ratios were shown as follows. At a sampling ratio of 1/10 (2766 data in training set and 24,635 in test set), the AUROC was highest in the sampling method of vector sum minimization, reaching 0.981 (95% confidence intervals (CIs): 0.980–0.983, *p* < 0.001). The second-highest AUROC was observed in stratified sampling at 0.965 (95% CIs: 0.963–0.967, *p* < 0.001), followed by simple random sampling at 0.956 (95% CIs: 0.954–0.958, *p* < 0.001), and the lowest AUROC was in vector sum maximization, registering 0.746 (95% CIs: 0.740–0.751, *p* < 0.001). (Figure 2A and Supplementary Table S1A). At a sampling ratio of 1/20 (1392 data in training set and 26,009 in test set), the AUROC remained highest in the vector sum minimization method at 0.963 (95% CIs: 0.961–0.965, *p* < 0.001). The other three methods ranked by AUROC values were stratified sampling, simple random sampling, and vector sum maximization. (Figure 2B and Supplementary Table S1B). At a sampling ratio of 1/30 (936 data in training set and 26,465 in test set) and 1/40 (706 data in training set and 26,695 in test set), the ranking of sampling methods by AUROC values maintained the same as in the previous two sampling ratios. (Figure 2C,D and Supplementary Table S1C,D).

All the semantic text mining models built by the samples drawn by four sampling methods could effectively identify VCF. Among the four sampling methods, all the models with the best predictive effect at the four different sampling ratios, noticed as the highest value of AUROC, were built by the samples drawn by the vector sum minimization sampling method. In contrast, the worst predictive effects were found in the predictive models built by the vector sum maximization sampling method, and the AUROC value is significantly lower than the other three sampling methods in any percentage sampling ratios. In addition, the predictive effect of the models was better in stratified sampling than simple random sampling, irrespective of the sampling ratios. (Supplementary Table S1).

## 4. Discussion

To the best of our knowledge, this is the first study that proposed using the sum of the document vector in groups clustered after HAC as a sampling basis for textual data and proved the vector sum minimization as an effective method to draw the little amount of critical samples to build the reliable semantic text mining models, which also became the first models to identify VCF from radiology reports. This study demonstrated that clustering textual data by the document vector was reasonably verified by the stratified sampling method based on the groups divided by HAC, and was shown to be having a better effect than the simple random sampling method for building predictive models. Under the basis of clustering the textual data by document vector, the following step drew samples relying on a vector sum relative to the cluster centers in each group that could be representative, clarified by the vector sum minimization as best and vector sum maximization as the worst method for the predictive effects of their own building models among four sampling methods. Both stratified by document vector and drawn by vector sum minimization were credible sampling methods, whereas the vector sum minimization sampling method was better.

### 4.1. The Importance of Text Mining Models

This study established the reliably semantic text mining models to identify the patients with VCF from the radiology reports of an X-ray. The under-diagnosis of VCF is a worldwide problem and the rates are reported as 34–50% [32]. Gehlbach et al. demonstrated that even when VCF was stated in radiology reports, only 17% of the hospitalized patients were mentioned. The diagnosis of VCF in discharging medical records resulted in a high possibility to miss further intervention and treatment [33]. Majumdar et al. revealed the treatment rate was 25% for osteoporosis of the patients with VCF reported by radiology reports [34]. The gaps from image exams to perform the treatment were caused by a lack of the awareness in VCF and the ambiguous descriptions in radiology reports [32,35]. Before applying the AI models, only 75.6% of the radiology reports of VCF were indicated “compression fracture” in this study. The semantic text mining models could detect not only the statement of “compression fracture” but also the descriptions with similar semantics as “compression fracture”. The identifying rates of VCF from radiology reports using the semantic text mining models built following the vector sum minimization sampling method were 95.1–98.8%, which indicates better performance as compared to only 64.5% using traditional keyword searching. Furthermore, the models can be helpful to rapidly detect the VCF patients from the radiological reports even decades ago. Therefore, the models can become a powerful AI-assisted system to actively remind the clinicians in identifying and treating the patients with VCF and potentially improving the treatment gap to prevent morbidity and mortality [11,12,13].

The technique of text mining was widely used in medicine for free-text data analyzing. The current studies showed the application of text mining in textual data such as electronic medical records to extract valuable information for medical decision support, disease risk prediction, drug reaction detection, personalized healthcare, and classification of diagnosis [36,37]. Huang et al. delivered a study to apply text mining in analyzing free-text medical records for assisting the treatment plan selections of smoking cessation [38]. Harpaz et al. demonstrated the use of text mining to extract the adverse drug events from multiple textual data and potentially improve pharmacovigilance [39]. Sugimoto et al. utilized text mining to extract the clinical information, such as anatomical terms, location terms, and size, from radiology reports of chest computed tomography [40]. Although text mining is a powerful tool in textual documents, the previous work showed the important step, manual annotations, before building the models. The advanced sampling methods for free-text data shown in this study could be a crucial technique to save the time by manually annotating a small size of partial data and minimizing the barriers of establishing text mining models.

### 4.2. The Importance of Sampling Methods in Textual Data

Manually labeling large amounts of samples to build the models was not efficient [20,21], whereas sampling methods were the solution drawn for the small-size representative sample [41]. However, there was a lack of studies focused on sampling methods in the textual data and proving the effectiveness in clinical practice. Our study proposed the sampling method based on the document vector and showed the significantly better predictive effect of the models in the stratified sampling setting compared with simple random sampling [42]. This result revealed that the document vector was the typical character of the textual data [43]. Moreover, the vector sum was calculated by the addition of the vector of each drawn sample relative to the cluster center. We proposed that minimizing the vector sum implied to the evenly distributed samples of their own groups in the vector space were drawn and became representative, whereas the maximization of the vector sum was the opposite setting to draw biased samples. About the comparing the predictive effects of the models among four settings of sampling method, vector sum minimization settings were demonstrated as significantly highest and vector sum maximization settings were the lowest compared with the other settings in any sampling ratios. These results supported that proposed vector sum minimization could be one of the reliable sampling methods in textual data to draw the critical representative samples. Additionally, implementing this concept in sampling image data based on the latent space could provide a viable approach [44]. In constructing predictive or classification models for images, manually labeling large amounts of data is inefficient. Drawing a small but representative subset of data is crucial and an efficient means to establish models through deep learning. Further investigation into developing a sampling method for image data based on the idea of vector sum minimization could be explored.

### 4.3. Implementation of Vector Sum Minimization

When applying sampling methods of vector sum minimization, all the possible permutations and combinations of the needed drawn samples in their groups were completed and then the samples’ combination with vector sum minimization was chosen. The document was converted into a 400-dimensional vector, a similar concept as 400 variables in each data, and there were around 600 data of each group in average. There were many possible permutations and combinations, and calculations combined with comparing the high dimensional vector sum in each dimension were complex missions. The time complexity estimated the amount of time taken by running an algorithm and should be considered for applying the sampling methods using a vector sum [45]. For the large database of textual documents with a higher dimensional vector, the time complexity was also higher resulting in the sampling methods becoming inefficient. In any case, if only considering “vector sum minimization” to draw the samples in each group, the final picking up samples may all be close to the cluster center, which means not well-distributed in the vector space and not well representative for their groups. For the above two problems, this study adopted a method to randomly draw a half number of the required samples in the first group. Moreover, drawing the other half of the samples that were closest to the mirror points of the taken samples in the first step was processed, and this method could possibly make the vector sum close to zero [46,47]. The mirror points mean the two points with the same vector value, relative to the cluster center, in each dimension but opposite in the plus–minus sign. This action reduced the time complexity in sampling and also ensured, to a certain extent, a well-distributed representation in the vector space. The first reason addressed the need to lower the time complexity during sampling. Additionally, this study demonstrated the achievement of the lowest vector sum among the three sampling methods with clustering, as shown in Table 1. The second reason aimed to ensure a reasonably even distribution throughout the vector space. If one were to calculate all possible permutations and combinations of vector sums, there was a potential risk of drawing most samples that are closely clustered around the center. This scenario may result in an insufficiently distributed sample set, failing to accurately reflect the real distribution within the vector space of that cluster.

### 4.4. Strengths

The strength of this study was establishing the sampling method as vector sum minimization in textual data to become the feasible way for efficiently drawing the smaller amount of critical representative data. This process could help the clinical staff save a huge amount of time manually labeling a large amount of data for building the semantic text mining models. For example, each labeling to the radiology reports for VCF cost 1 min, whereas applying the sampling at the ratio of 1/40 from 30,000 textual data could save 39,250 min. Sampling the critical representative data to become the training set would make the better text mining models in extracting the information correctly. The sampling method of vector sum minimization might be a strong tool not only in radiology reports but also in other textual data in medicine.

The semantic text mining models to identify VCF employed in the FLS of E-Da Hospital met Standard 4 of CTF^®^ [14]. Every radiology free-text report for spine X-ray, computed tomography or magnetic resonance imaging were automatically reviewed by the models to recognize VCF and presented the list of the patients with VCF for the healthcare staff. The other models for identifying femoral fracture and osteoporosis were also built in a similar way as the models for determining VCF, utilizing the vector sum minimization sampling method. Under the assistance of the models, a total of 5746/245,764 reports of dual-energy X-ray absorptiometry/imaging exam of the spine and hip were analyzed in each year on average. An average of 9634/11,603/1047 patients with VCF/femoral fracture/osteoporosis were identified each year, and the recognition accuracy rate was 96.3%. These achievements were certified as 100% CTF^®^ Gold Award by IOF [24].

### 4.5. Limitations

This study has the following limitations. First, the enrolling textual data are in English; however, the applications of the vector sum minimization sampling method in other languages require further investigation. Second, this study was focused on VCF with 40.7% positive in enrolling data since the prevalence rate of the disease was the influent factor of text mining models [48]. Third, this study compared the performance of sampling methods across four sampling ratios. We observed a decline in performance with a reduction in the number of sampled data for the training set. However, an analysis of the minimum sample size required for the training set, which is crucial and practical, was not investigated in this study. Fourth, the LSTM algorithm was utilized to construct all the semantic text mining models. However, exploring advancements in neural network architectures could potentially enhance the performance of semantic text mining models, and other algorithms were not tested in this study. Fifth, although the sampling method performed well in our dataset across four sampling ratios, external validation is still necessary to assess its generalizability.

## 5. Conclusions

This study confirmed the advanced sampling method, especially the vector sum minimization, in textual data that can efficiently build the semantic text mining models by drawing a small amount of critical representative samples and saving the time for labeling the large amount of samples. By efficiently building the models under the assistance of the sampling method, extracting the usefully clinical information could be achieved for further statistical analysis and clinical care such as identifying the undertreated patients who were diagnosed in textual reports without treatment. Meanwhile, the responsible reporters for the medical exam do not need to change their behaviors for typing the textual reports because the mining models could automatically extract the interesting information simultaneously.

## Figures and Tables

**Table 1:** Vector sum presented by Euclidean distance in each sampling ratio and method.

Sampling Ratio	Sampling Method	Euclidean Distance of Each Cluster	*p* Value
1/10 (*N* = 2766)	Vector sum minimization	2.9 ± 2.3	<0.001
	Vector sum maximization	19.5 ± 26.1	
	Stratified sampling	12 ± 10.3	
	Simple random sampling	N/A	N/A
1/20 (*N* = 1392)	Vector sum minimization	4.0 ± 2.6	<0.001
	Vector sum maximization	22.8 ± 32.1	
	Stratified sampling	9.7 ± 7.8	
	Simple random sampling	N/A	N/A
1/30 (*N* = 936)	Vector sum minimization	4.1 ± 3.1	<0.001
	Vector sum maximization	28.8 ± 38.0	
	Stratified sampling	14.4 ± 12.5	
	Simple random sampling	N/A	N/A
1/40 (*N* = 706)	Vector sum minimization	5.9 ± 4.6	<0.001
	Vector sum maximization	42.8 ± 54.8	
	Stratified sampling	18.4 ± 18.9	
	Simple random sampling	N/A	N/A

N/A: Non-applicable. Data are expressed as means ± standard deviation.

## Data Availability

The datasets analyzed during the current study are available from the corresponding author on reasonable request.

## Supplementary Materials

The following supporting information can be downloaded at: https://www.mdpi.com/article/10.3390/diagnostics14020137/s1, Figure S1: Algorithm of building semantic text mining models using LSTM network; Figure S2: Applying hierarchical agglomerative clustering by document vectors to divide into 46 groups expressed in 2-dimension; Table S1: Demonstration and comparison of the predictive effects of semantic text mining models built using the samples drawn by different sampling methods (A) sampling ratio of 1/10 (B) sampling ratio of 1/20 (C) sampling ratio of 1/30, and (D) sampling ratio of 1/40.

## References

[1] Miner G., Elder J., Fast A., Hill T., Nisbet R., Delen D. (2012). Practical Text Mining and Statistical Analysis for Non-Structured Text Data Applications.

[2] Sturgis P. (2004). The effect of coding error on time use surveys estimates. J. Off. Stat..

[3] Brodley C.E., Friedl M.A. (1999). Identifying mislabeled training data. J. Artif. Intell. Res..

[4] Nadkarni P.M., Ohno-Machado L., Chapman W.W. (2011). Natural language processing: An introduction. J. Am. Med. Inform. Assoc..

[5] Kao A., Poteet S.R. (2007). Natural Language Processing and Text Mining.

[6] Blumenthal D., Tavenner M. (2010). The “Meaningful Use” Regulation for Electronic Health Records. N. Engl. J. Med..

[7] Mahmoudi E., Kamdar N., Kim N., Gonzales G., Singh K., Waljee A.K. (2020). Use of electronic medical records in development and validation of risk prediction models of hospital readmission: Systematic review. BMJ.

[8] Cook D.J., Guyatt G.H., Adachi J.D., Clifton J., Griffith L.E., Epstein R.S., Juneper E.F. (1993). Quality of life issues in women with vertebral fractures due to osteoporosis. Arthritis Rheum. Off. J. Am. Coll. Rheumatol..

[9] Center J.R., Nguyen T.V., Schneider D., Sambrook P.N., Eisman J.A. (1999). Mortality after all major types of osteoporotic fracture in men and women: An observational study. Lancet.

[10] Schousboe J.T. (2016). Epidemiology of Vertebral Fractures. J. Clin. Densitom..

[11] Resch A., Schneider B., Bernecker P., Battmann A., Wergedal J., Willvonseder R., Resch H. (1995). Risk of vertebral fractures in men: Relationship to mineral density of the vertebral body. AJR. Am. J. Roentgenol..

[12] Lindsay R., Silverman S.L., Cooper C., Hanley D.A., Barton I., Broy S.B., Licata A., Benhamou L., Geusens P., Flowers K. (2001). Risk of new vertebral fracture in the year following a fracture. JAMA.

[13] Francis R., Baillie S., Chuck A., Crook P., Dunn N., Fordham J., Kelly C., Rodgers A. (2004). Acute and long-term management of patients with vertebral fractures. QJM.

[14] Marsh D., Åkesson K., Beaton D., Bogoch E., Boonen S., Brandi M.-L., McLellan A., Mitchell P., Sale J., Wahl D. (2011). Coordinator-based systems for secondary prevention in fragility fracture patients. Osteoporos. Int..

[15] Adler-Milstein J., Everson J., Lee S.Y.D. (2015). EHR adoption and hospital performance: Time-related effects. Health Serv. Res..

[16] Grundmeier R.W., Masino A.J., Casper T.C., Dean J.M., Bell J., Enriquez R., Deakyne S., Chamberlain J.M., Alpern E.R., The Pediatric Emergency Care Applied Research Network (2016). Identification of Long Bone Fractures in Radiology Reports Using Natural Language Processing to support Healthcare Quality Improvement. Appl. Clin. Inform..

[17] Chandran M. (2013). Fracture Liaison Services in an open system: How was it done? what were the barriers and how were they overcome?. Curr. Osteoporos. Rep..

[18] Senay A., Delisle J., Banica A., Laflamme G.Y., Leduc S., Mac-Thiong J.-M., Ranger P., Rouleau D., Fernandes J.C. (2018). Barriers to the identification of fragility fractures for secondary fracture prevention in an orthopaedic clinic-based fracture liaison service: A prospective cohort study. Curr. Orthop. Pract..

[19] Kotsiantis S.B., Zaharakis I., Pintelas P. (2007). Supervised machine learning: A review of classification techniques. Emerg. Artif. Intell. Appl. Comput. Eng..

[20] Singh R.P., Hom G.L., Abramoff M.D., Campbell J.P., Chiang M.F., AAO Task Force on Artificial Intelligence (2020). Current Challenges and Barriers to Real-World Artificial Intelligence Adoption for the Healthcare System, Provider, and the Patient. Transl. Vis. Sci. Technol..

[21] Miller D.D., Brown E.W. (2018). Artificial intelligence in medical practice: The question to the answer?. Am. J. Med..

[22] Namee B.M., Cunningham P., Byrne S., Corrigan O.I. (2002). The problem of bias in training data in regression problems in medical decision support. Artif. Intell. Med..

[23] Cochran W.G. (2007). Sampling Techniques.

[24] Hung W., Yang C., Cheng W., Wu C. (2019). Revisit three “I” model: A novel five “I” model of fracture liaison service. Osteoporos. Int..

[25] Le Q., Mikolov T. Distributed representations of sentences and documents. Proceedings of the International Conference on Machine Learning.

[26] Naeem A., Rehman M., Anjum M., Asif M. (2019). Development of an efficient hierarchical clustering analysis using an agglomerative clustering algorithm. Curr. Sci..

[27] Liberti L., Lavor C. (2017). Euclidean Distance Geometry: An Introduction.

[28] Skovajsová L. (2017). Long short-term memory description and its application in text processing. Proceedings of the 2017 Communication and Information Technologies (KIT).

[29] DeLong E.R., DeLong D.M., Clarke-Pearson D.L. (1988). Comparing the areas under two or more correlated receiver operating characteristic curves: A nonparametric approach. Biometrics.

[30] Bewick V., Cheek L., Ball J. (2004). Statistics review 13: Receiver operating characteristic curves. Crit. Care.

[31] Bradley A.P. (1997). The use of the area under the ROC curve in the evaluation of machine learning algorithms. Pattern Recognit..

[32] Panda A., Das C.J., Baruah U. (2014). Imaging of vertebral fractures. Indian J. Endocrinol. Metab..

[33] Gehlbach S.H., Bigelow C., Heimisdottir M., May S., Walker M., Kirkwood J.R. (2000). Recognition of vertebral fracture in a clinical setting. Osteoporos. Int..

[34] Majumdar S.R., Kim N., Colman I., Chahal A.M., Raymond G., Jen H., Siminoski K.G., Hanley D.A., Rowe B.H. (2005). Incidental vertebral fractures discovered with chest radiography in the emergency department: Prevalence, recognition, and osteoporosis management in a cohort of elderly patients. Arch. Intern. Med..

[35] Lenchik L., Rogers L.F., Delmas P.D., Genant H.K. (2004). Diagnosis of osteoporotic vertebral fractures: Importance of recognition and description by radiologists. AJR Am. J. Roentgenol..

[36] Pereira L., Rijo R., Silva C., Martinho R. (2015). Text Mining Applied to Electronic Medical Records: A Literature Review. Int. J. E Health Med. Commun..

[37] Sun W., Cai Z. (2018). Data Processing and Text Mining Technologies on Electronic Medical Records: A Review. J. Health Eng..

[38] Huang H.-L., Hong S.-H., Tsai Y.-C. (2020). Approaches to text mining for analyzing treatment plan of quit smoking with free-text medical records: A PRISMA-compliant meta-analysis. Medicine.

[39] Harpaz R., Callahan A., Tamang S., Low Y., Odgers D., Finlayson S., Jung K., LePendu P., Shah N.H. (2014). Text mining for adverse drug events: The promise, challenges, and state of the art. Drug Saf..

[40] Sugimoto K., Takeda T., Oh J.-H., Wada S., Konishi S., Yamahata A., Manabe S., Tomiyama N., Matsunaga T., Nakanishi K. (2021). Extracting clinical terms from radiology reports with deep learning. J. Biomed. Inform..

[41] Li D.-C., Hu S.C., Lin L.-S., Yeh C.-W. (2017). Detecting representative data and generating synthetic samples to improve learning accuracy with imbalanced data sets. PLoS ONE.

[42] Sedgwick P. (2013). Stratified cluster sampling. BMJ.

[43] Kowsari K., Meimandi K.J., Heidarysafa M., Mendu S., Barnes L., Brown D. (2019). Text classification algorithms: A survey. Information.

[44] Lassance C., Gripon V., Ortega A. (2021). Representing deep neural networks latent space geometries with graphs. Algorithms.

[45] Jonsson P., Lagerkvist V. (2017). An initial study of time complexity in infinite-domain constraint satisfaction. Artif. Intell..

[46] Riesen K., Bunke H. (2009). Graph classification based on vector space embedding. Int. J. Pattern Recognit. Artif. Intell..

[47] Hao P.-Y., Chiang J.-H., Tu Y.-K. (2007). Hierarchically SVM classification based on support vector clustering method and its application to document categorization. Expert Syst. Appl..

[48] Lobo J.M., Jiménez-Valverde A., Real R. (2008). AUC: A misleading measure of the performance of predictive distribution models. Glob. Ecol. Biogeogr..

