# Supplementary material for: Advanced Sampling Technique in Radiology Free-Text Data for Efficiently Building Text Mining Models by Deep Learning in Vertebral Fracture
Source: Diagnostics (Basel). 2024 Jan 8;14(2):137. doi: 10.3390/diagnostics14020137 (PMC10814913; doi:10.3390/diagnostics14020137)

Supplementary tables

Supplementary table S1. Demonstration and comparison of the predictive effects of semantic text mining models built using the samples drawn by different sampling methods (A) sampling ratio of 1/10 (B) sampling ratio of 1/20 (C) sampling ratio of 1/30, and (D) sampling ratio of 1/40

Supplementary table S1(A). Sampling ratio of 1/10

	Sampling ratio 1/10 (N = 2,766 in training set, N = 24,635 in test set)			
	Vector sum minimization	Vector sum maximization	Stratified sampling	Simple random sampling
AUROC (95% CIs)	0.981 (0.980 to 0.983)*	0.746 (0.740 to 0.751)*	0.965 (0.963 to 0.967)*	0.956 (0.954 to 0.958)*
Difference in AUROC (95% CIs)				
Versus simple random sampling	0.025 (0.023 to 0.027)*	−0.210 (−0.205 to −0.216)*	0.009 (0.008 to 0.010)*	-
Versus stratified sampling	0.016 (0.014 to 0.018)*	−0.220 (−0.214 to −0.225)*	-	-
Versus vector sum maximization	0.236 (0.230 to 0.242)*	-	-	-

Supplementary table S1(B). Sampling ratio of 1/20

	Sampling ratio 1/20 (N = 1,392 in training set, N = 26,009 in test set)			
	Vector sum minimization	Vector sum maximization	Stratified sampling	Simple random sampling
AUROC (95% CIs)	0.963 (0.961 to 0.965)*	0.684 (0.678 to 0.690)*	0.916(0.913 to 0.919)*	0.889 (0.885 to 0.893)*
Difference in AUROC (95% CIs)				
Versus simple random sampling	0.074 (0.071 to 0.078)*	−0.205 (−0.199 to −0.211)*	0.027 (0.025 to 0.029)*	-
Versus stratified sampling	0.047 (0.044 to 0.050)*	−0.232 (−0226 to −0.239)*	-	-
Versus vector sum maximization	0.279 (0.272 to 0.285)*	-	-	-

Supplementary table S1(C). Sampling ratio of 1/30

	Sampling ratio 1/30 (N = 936 in training set, N = 26,465 in test set)			
	Vector sum minimization	Vector sum maximization	Stratified sampling	Simple random sampling
AUROC (95% CIs)	0.907 (0.904 to 0.911)*	0.638 (0.632 to 0.643)*	0.843 (0.839 to 0.847)*	0.815 (0.810 to 0.819)*
Difference in AUROC (95% CIs)				
Versus simple random sampling	0.093 (0.089 to 0.097)*	−0.177 (−0.169 to −0.185)*	0.028 (0.025 to 0.032)*	-
Versus stratified sampling	0.064 (0.062 to 0.067)*	−0.205 (−0.198 to −0.213)	-	-
Versus vector sum maximization	0.270 (0.263 to 0.277)*	-	-	-

Supplementary table S1(D). Sampling ratio of 1/40

	Sampling ratio 1/40 (N = 706 in training set, N = 26,695 in test set)			
	Vector sum minimization	Vector sum maximization	Stratified sampling	Simple random sampling
AUROC (95% CIs)	0.895 (0.891 to 0.899)*	0.633 (0.628 to 0.639)*	0.820 (0.816 to 0.825)*	0.802 (0.797 to 0.807)*
Difference in AUROC (95% CIs)				
Versus simple random sampling	0.093 (0.089 to 0.097)*	−0.168 (−0.161 to −0.176)*	0.019 (0.014 to 0.023)*	-
Versus stratified sampling	0.075 (0.071 to 0.078)*	−0.187 (−0.180 to −0.194)*	-	-
Versus vector sum maximization	0.262 (0.254 to 0.269)*	-	-	-

*P value < 0.001

‡AUROC: area under the receiver operating characteristics

†95% CIs: 95% confidence intervals

Supplementary Fig S1. Algorithm of building semantic text mining models using LSTM network

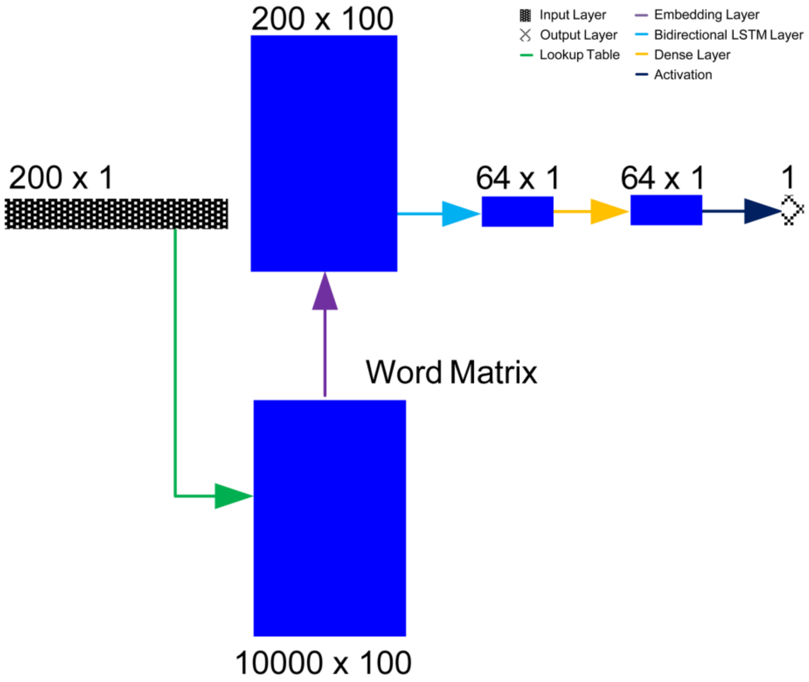

*LSTM: long short-term memory

#The settings of the process were (1) the length of input sequences (input dimensions): 200x1; (2) the word matrix: 10000x100; (3) the feature extraction: 200x100; (4) mapping: 64x1 (5) output dimension: 1

Supplementary Fig S2. Applying hierarchical agglomerative clustering by document vectors to divide into 46 groups expressed in 2-dimension

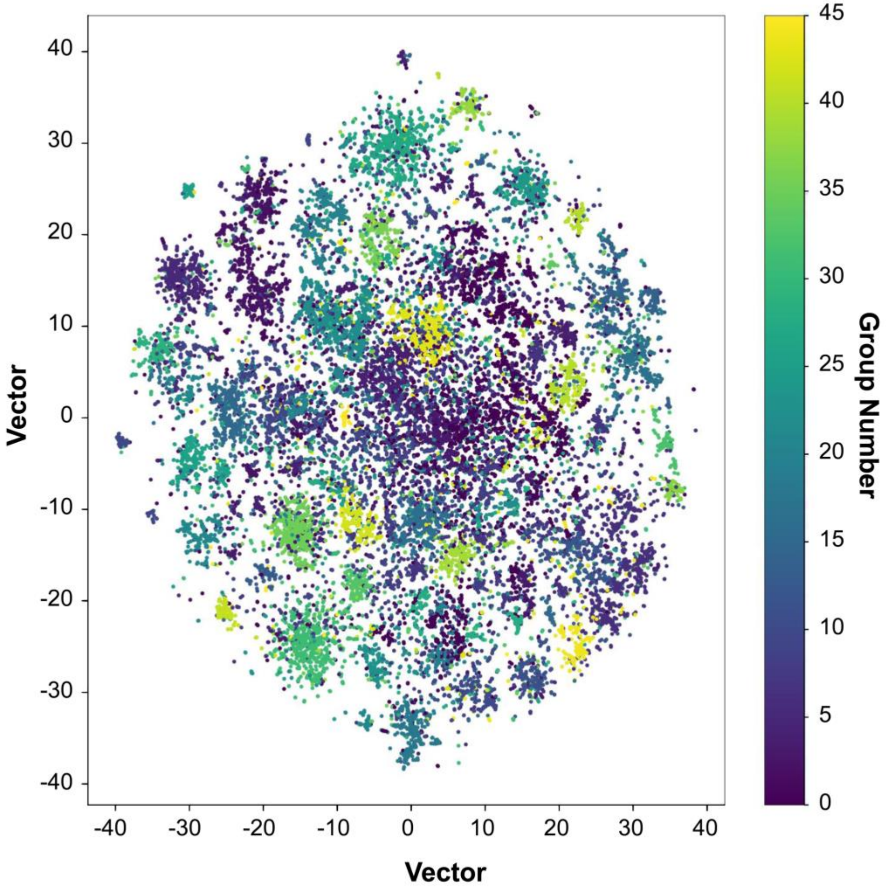

Supplement: Supplementary file 1 [file diagnostics-14-00137-s001.zip › supplementary information.pdf]
